# Association of HFE common mutations with Parkinson's disease, Alzheimer's disease and mild cognitive impairment in a Portuguese cohort

**DOI:** 10.1186/1471-2377-6-24

**Published:** 2006-07-06

**Authors:** Rita J Guerreiro, Jose M Bras, Isabel Santana, Cristina Januario, Beatriz Santiago, Ana S Morgadinho, Maria H Ribeiro, John Hardy, Andrew Singleton, Catarina Oliveira

**Affiliations:** 1 Laboratory of Neurogenetics, National Institute on Aging, National Institutes of Health, Bethesda, USA; 2 Center for Neuroscience and Cell Biology, University of Coimbra, Coimbra, Portugal; 3 Neurology Service, University of Coimbra Hospital, Coimbra, Portugal

## Abstract

**Background:**

Pathological brain iron deposition has been implicated as a source of neurotoxic reactive oxygen species in Alzheimer (AD) and Parkinson diseases (PD). Iron metabolism is associated with the gene hemochromatosis (*HFE *Human genome nomenclature committee ID:4886), and mutations in *HFE *are a cause of the iron mismetabolism disease, hemochromatosis. Several reports have tested the association of *HFE *variants with neurodegenerative diseases, such as AD and PD with conflicting results.

**Methods:**

Genotypes were analysed for the two most common variants of *HFE *in a series of 130 AD, 55 Mild Cognitive Impairment (MCI) and 132 PD patients. Additionally, a series of 115 healthy age-matched controls was also screened.

**Results:**

A statistically significant association was found in the PD group when compared to controls, showing that the presence of the C282Y variant allele may confer higher risk for developing the disease.

**Conclusion:**

Taken together these results suggest that the common variants in *HFE *may be a risk factor for PD, but not for AD in the Portuguese population.

## Background

Classic Hemochromatosis is an autosomal recessive disorder that is associated with a deregulation of the iron metabolism [1]. Clinical features often include cirrhosis of the liver, diabetes, hypermelanotic pigmentation of the skin, and heart failure [2]. Hemochromatosis is most often caused by mutations in the gene *HFE *on chromosome 6p21.3. The most common mutation, C282Y, was initially found in a subset of patients with hereditary hemochromatosis, in a total of 83% of all individuals. A second mutation, H63D, was also described, although the clinical effects of this modification are clearly more limited. However, about 1 to 2 percent of individuals with compound heterozygous *HFE *mutations appear to be at risk for hemochromatosis [3].

Alzheimer's disease is the most common late-onset neurodegenerative disorder. While several studies have tried to unveil the precise mechanisms underlying the etiology of typical sporadic AD, these remain largely unknown. Nonetheless, several studies have reported that oxidative stress may be implicated in the pathogenesis of this condition [4,5]. Oxidative damage in AD brain may be due, at least partially, to the increased deposition of redox-active iron, which is an important generator of reactive oxygen species (ROS) [6]. *HFE *mutations have been associated with different stages of dementia (Braak stages) and increased oxidative stress, thus a study including MCI patients is of relevance [7]. However this remains only a speculation, as the mechanisms governing pathological brain iron deposition in AD are still unidentified.

Parkinson's disease is the second most common form of neurodegenerative disease, characterized clinically by resting tremor, muscular rigidity, bradykinesia, and postural instability. Post-mortem examinations of PD brains and magnetic resonance imaging of PD patients have revealed increased iron contents in the substantia nigra [8]. The cause for this deposition is unclear; however it has been speculated to result in overproduction of free radicals, which in turn, may cause lipid peroxidation, protein and nucleic acid oxidation [9].

Previous studies assessing the effect of *HFE *variants on the onset of PD and AD have been contradictory [7,10-12]. Thus, to ascertain if *HFE *mutations are a risk factor for the development of these diseases, we conducted a genetic screening for the most common *HFE *mutations in two series of patients and in a healthy control group.

## Methods

### Subjects

A total of 428 individuals were screened for two mutations in the HFE gene: C282Y (rs1800562) and H63D (rs1799945).

The first series of patients integrated AD and MCI patients. The diagnosis of probable Alzheimer's disease was made in accordance with criteria defined by the Diagnostic and Statistical Manual, revision 4 (DSM-IV) [13] and the guidelines of the National Institute of Neurological Disorders and Stroke, and the Alzheimer's Disease and Related Disorders Association (NINCDS-ADRDA) [14]. All participants were assessed with the Mini Mental-State Examination (MMSE) [15] and the Clinical Dementia Rating (CDR) [16] scales. The diagnosis of MCI was made in accordance with criteria defined by Petersen [17]. AD patients were selected from a consecutive clinic case series of those who gave permission for sampling (over 90% of cases consent for blood sampling), collected by neurologists at the University of Coimbra Hospital. Selection was performed to include patients with a negative familial history and a late age at onset for the disease (≥ 65 years). This group included 130 patients (79 females and 51 males) with mean ages of 75 ± 5 years and mean age at onset of 71,5 ± 4,9 years. In this series were also included 55 MCI patients (32 females and 23 males) with mean ages of 69,5 ± 9,6 and mean age at onset of 67,5 ± 9,4 years.

A total of 132 PD patients were selected according to the United Kingdom Parkinson's Disease Society Brain Bank Clinical Diagnostic Criteria (UK PDS Brain Bank) [18]. Patients comprised a consecutive clinic based cohort (over 90% of cases consent for blood sampling), diagnosed by a movement disorder specialist at the movement disorder clinic of the University of Coimbra Hospital. This series included 62 males and 70 females, with mean of ages of 66,7 ± 10,7 years, and mean age at onset of 57,2 ± 12,0 years. From these, 28 patients presented with a positive family history for PD, while the remaining 104 showed no evidence of family history for PD or any form of parkinsonism.

The control group included 115 healthy controls with a mean age of 70,7 ± 10,3 years, 38 males and 77 females. All subjects were examined by a neurologist and were free of any clinical signs or symptoms of neurodegeneration. This group comprised mainly spouses of patients and caregivers that were accompanying patients to the clinic.

All individuals included in this study are Caucasian with an apparent Portuguese ancestry. The study was submitted to the Ethics board of the University Hospital of Coimbra and all the subjects involved gave their informed consent.

### Genotyping

Genomic DNA was isolated from whole blood by means of standard procedures and the samples were genotyped for the *HFE *mutations C282Y and H63D using the polymerase chain reaction (PCR) technique with subsequent restriction and gel electrophoresis, as previously described [12]. Similarly, *APOE *genotypes were assessed by a PCR-based methodology, as previously described [19].

### Statistical analysis

Observed genotype distributions were compared with those expected by cross-tabulation and analyzed using Chi-square and Fisher Exact-tests. Means of quantitative variables were compared using Student's *t*-test. Kaplan-Meier (KM) survival analysis was used to analyze the effects of the *HFE *mutations on the age of AD, PD and MCI onset. The log-rank test was employed to determine whether genotype-specific survival functions were significantly different from one another. All tests were interpreted at the 0,05 level of significance. All statistical analyses were performed with the SPSS package, version 10.0 (SPSS, Chicago, IL, USA).

## Results

To test the association between the presence of the C282Y and H63D mutations and the development of AD or PD, we screened these series of patients and a series of healthy controls. The genotypes in these cohorts were at or near Hardy-Weinberg equilibrium.

There was no significant difference in genotype or allele frequencies in the AD or MCI groups, compared to controls. Analysis of the genotypes in the PD series revealed a significant overrepresentation of 282Y carriers and of the 282Y allele compared to controls (p = 0.01) (Tables 1 and 2). There was a statistically significant difference in the number of 282Y carriers between the AD and PD groups (p = 0.01). No other associations were found in any of the series we studied (Tables 1 and 2).

**Figure 1:**
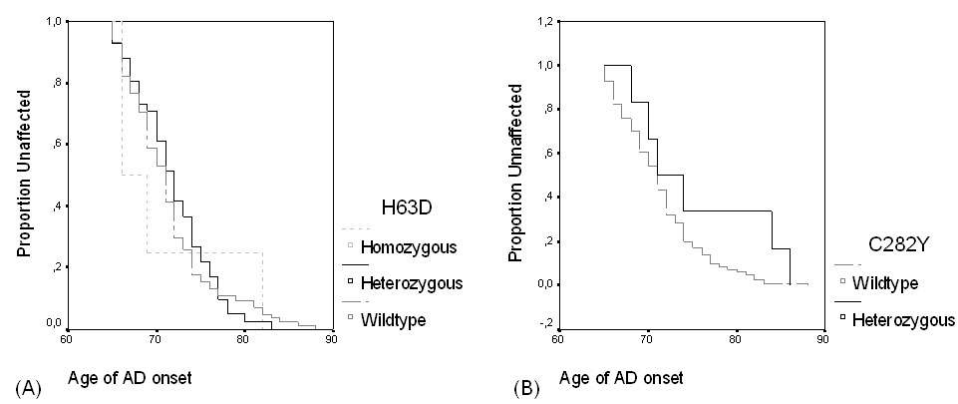
**Kaplan-Meier survival curves indicating the effect of H63D and C282Y mutations on age of AD onset**. (A) There are no statistically significant differences in the age at onset of AD between wild type, heterozygous and homozygous patients for H63D mutation (χ^2^(2df) = 0.14, *P *= 0.93). (B) There are no statistically significant differences in the age at onset of the disease between wild type, heterozygous and homozygous patients for C282Y mutation (χ^2^(1df) = 3.08, *P *= 0.08).

**Figure 2:**
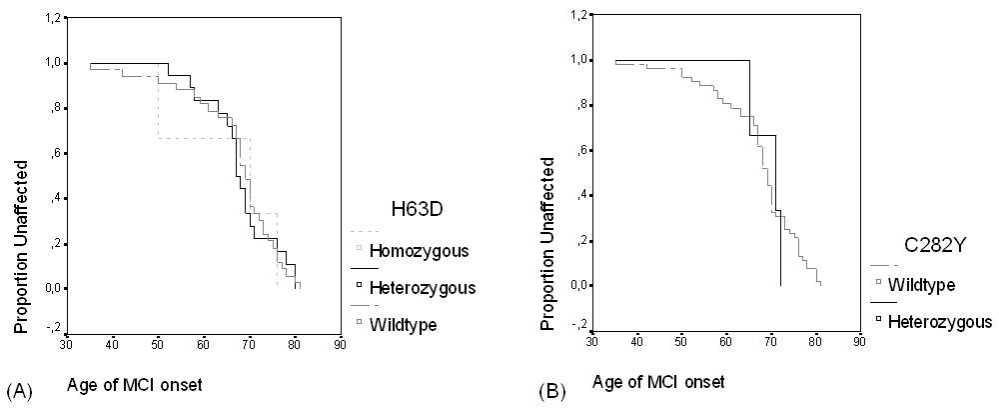
**Kaplan-Meier survival curves indicating the effect of H63D (A) and C282Y (B) mutations on age of MCI onset**. There are no statistically significant differences in the age at onset of MCI between wild type, heterozygous and homozygous patients for H63D mutation (χ^2^(2df) = 0.04, *P *= 0.98). (B) There are no statistically significant differences in the age at onset of MCI between wild type, heterozygous and homozygous patients for C282Y mutation (χ^2^(1df) = 0.10, *P *= 0.76).

**Figure 3:**
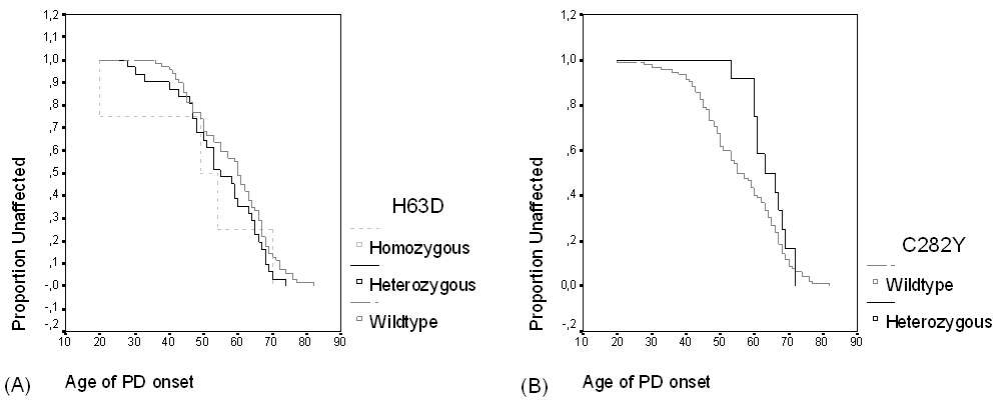
**Kaplan-Meier survival curves indicating the effect of H63D and C282Y mutations on age of PD onset**. (A) There are no statistically significant differences in the age at onset of PD between wild type, heterozygous and homozygous patients for H63D mutation (χ^2^(2df) = 2.4, P = 0.30). (B) There are no statistically significant differences in the age at onset of PD between wild type, heterozygous and homozygous patients for C282Y mutation (χ^2^(1df) = 1.66, P = 0.20).

The outcome of the genetic mutations studied may also affect the age at onset of the studied disorders. Therefore we used Kaplan-Meier survival curves to determine this outcome. We failed to find any association between the mutations studied and the age at onset of PD, AD or MCI, as can be seen in Figures 1, 2 and 3.

The effect of many gene polymorphisms may be altered by the presence or absence of the ApoE4 allele. Stratifying based on E4 status did not reveal any significant differences in C282Y and H63D mutation frequency in AD patients compared to controls (Table 3).

We did not observe any differences between groups when the data were analysed according to gender (data not shown).

## Discussion

Our results suggest that C282Y and H63D variants of *HFE *do not contribute significantly to the risk of developing AD or MCI, in the Portuguese population. These data are consistent with previous studies [12,21] but contradictory to others such as those by Moalem and colleagues who reported that HFE mutations predisposed to familial AD in ApoE E4 negative males [22] and data from Pulliam et al. that suggested that *HFE *mutations were associated with increased oxidative stress and Braak AD stage [7]. The latter study was the primary impetus behind us studying these variants in MCI, a recognised prodromal stage of AD. Other studies demonstrated the potential association between the mutant H63D allele and the age at onset of AD [11,23]. Sampietro and colleagues reported that in an Italian sample, where the C282Y mutation is very uncommon, onset of AD occurred about 5 years earlier in subjects carrying one or more copies of the H63D mutation, independently of gender. In patients under 70 years at disease onset, the incidence of the H63D mutation was five times higher than in those over 80 years at onset of the disease [24]. These studies suggest that not just homozygosity but also heterozygosity for the main HFE mutations may influence AD pathogenesis. In our sample no association between the studied mutations (in homozygosity or heterozygosity) and the age at onset of AD, MCI or PD was found.

The lack of association between genetic variability in *HFE *and AD in the current study may be related to one or more of several factors: first, the present results may represent a false negative finding, driven in part by the low sample numbers; second, the role of *HFE *variants in risk for disease may vary between different populations (ie genetic background); third, as discussed below, the variants studied here may not be disease causing, but in linkage disequilibrium with disease causing mutations, thus discordant results will be seen in different populations.

The data presented here show a significant increase of the prevalence of 282Y carriers in the PD cohort compared to controls. A previous study examining the relationship between *HFE *variants and PD reported an opposite effect to the data presented here: the authors presented data suggesting that individuals with C282Y mutation have a decreased risk of developing PD [11], in contrast an additional study suggests no role of *HFE *variants in risk for PD [20] and recent work describes a positive relationship between the 282Y variant and PD risk, consistent with the data presented in the current study [10].

The discordant results may be explained by several factors: first, the results of the current study and those of Dekker and colleagues represent false positive findings; second, the results of Buchannan and colleagues represent false positive findings; third, 282Y is not a causal variant but is in linkage disequilibrium with another variant that underlies disease risk. The degree and direction of a disease association when genotyping what is in effect a tagging SNP, are both sensitive to the structure and content of a given block of linkage disequilibrium; these factors are both potentially different between populations. The observation that the 282Y allele is overrepresented in the PD cohort compared to the AD cohort demonstrates explicitly the main findings of this paper; that this variant may infer risk for PD, but not AD in the Portuguese population. While it is tempting to speculate that differences in iron handling may differentiate the molecular underpinnings of these two disorders, the current data is too far removed from this mechanistically and too preliminary to make this a convincing argument. The infrequency of C282Y mutations obviously limits the statistical power of this analysis, thus, studies in larger samples from diverse populations are needed to clarify the relationship between variability in *HFE *and PD The small number of individuals in this study makes an ultimate assessment of the biological and genetic significance of these data clearly impossible. Thus we have analysed all previous studies published so far on this subject, in order to perform a meta-analysis of the data, and hopefully shed some light on these mechanisms.

Regarding AD, a total of five studies has been published aiming to find an association of these HFE mutations with the disease. No statistically significant differences were found in any of the studies. The combined data set can be observed in Tables 4 and 5.

As for PD, two studies have described a positive association between the C282Y variation and the disease [10,11], as can be seen in Tables 6 and 7. However, the study of Buchanan et al. includes siblings in the control group, which may have biased the results. As for the work of Dekker et al., the association was only evident in one of the studied cohorts: the Rotterdam study.

When looking at all the results published so far, there is no statistically significant association between any of the variations and the development of any of the diseases (Tables 4,5,6,7).

## Conclusion

In conclusion we present data that suggests genetic variability in *HFE *may be a risk factor for PD. The rarity of *HFE *282Y limits the statistical power of this analysis, thus studies in larger samples and in diverse cohorts are needed to make clarify the relation between variability in *HFE *and PD.

## Competing interests

The author(s) declare that they have no competing interests.

## Authors' contributions

RJG and JMB performed the genotyping, statistical analysis, and drafted the manuscript. MHR, IS, BS, CJ and ASM contributed to collecting materials. JH, AS and CRO participated in the study design and coordination, together with drafting the manuscript. All authors read and approved the final manuscript.

## Pre-publication history

The pre-publication history for this paper can be accessed here:



## Figures and Tables

**Table 1:** Genotype frequencies for HFE mutations in controls, AD, PD and MCI patients

	C282Y			p	H63D			*p*
			
	AA	GA	GG		GG	CG	CC	
Controls (n = 115)	0	5 (4.3%)	110 (95.7%)		2 (1.7%)	39 (33.9%)	74 (64.3%)	
AD (n = 130)	0	6 (4.6%)	124 (95.4%)	0.92	4 (3.1%)	41 (31.5%)	85 (65.4%)	0.76
MCI (n = 55)	0	3 (5.5%)	52 (94.5%)	0.75	3 (5.6%)	18 (33.3%)	33 (61.1%)	0.39
PD (n = 132)	0	18 (13.6%)	114 (86.4%)	0.01*	5 (3.8%)	38 (28.8%)	89 (67.4%)	0.47

*p < 0.05 (Statistically significant)

**Table 2:** Allelic frequencies for HFE mutations in controls, AD, PD and MCI patients

	*C282Y*		*p*	*H63D*		*p*
	A	G		G	C	

Controls (n = 115)	5 (2.2%)	225 (97.8%)		43 (18.7%)	187 (81.3%)	
AD (n = 130)	6 (2.3%)	254 (97.7%)	0.92	49 (18.8%)	211 (81.2%)	0.97
MCI (n = 55)	3 (2.7%)	107 (97.3%)	0.75	24 (22.2%)	84 (77.8%)	0.45
PD (n = 132)	18 (6.8%)	246 (93.2%)	0.01*	48 (18.2%)	216 (81.8%)	0.88

*p < 0.05 (Statistically significant)

**Table 3:** Genotypes associated with C282Y and H63D in AD patients with (ApoE4+) and without (ApoE4-) ApoE4 allele

	C282Y			p	H63D			*p*
			
	AA	GA	GG		GG	CG	CC	
ApoE4(+)	0	7.0%	93.0%		1.8%	29.8%	68.4%	
ApoE4(-)	0	2.9%	97.1%	0.25	2.9%	31.4%	65.7%	0.90

**Table 4:** Meta-analysis of the C282Y variation in the five published studies regarding Alzheimer's disease

**AD**	C282Y						
	**Patients**			**Controls**			

**Author**	wt/wt	wt/mut	mut/mut	wt/wt	wt/mut	mut/mut	*p*

Moalem, S. [22]	23 (88.5%)	3 (11.5%)	0	87 (92.5%)	7 (7.5%)	0	
Candore, G. [21]	121 (98.4%)	2 (1.6%)	0	151 (99.3%)	1 (0.7%)	0	
Berlin, D. [12]	95 (95%)	5 (5%)	0	90 (90%)	10 (10%)	0	
Sampietro, M. [24]	103 (96.3%)	4 (3.7%)	0	95 (96%)	4 (4%)	0	
Robson, KJ. [25]	161 (84.3%)	30 (15.7%)	0	237 (88.1%)	31 (11.5%)	1 (0.4%)	
**Total**	503	44	0	660	53	1	0.63

**Table 5:** Meta-analysis of the H63D variation in the five published studies regarding Alzheimer's disease

**AD**	H63D						
	**Patients**			**Controls**			

**Author**	wt/wt	wt/mut	mut/mut	wt/wt	wt/mut	mut/mut	*p*

Moalem, S. [22]	20 (76.9%)	6 (23.1%)	0	67 (72%)	23 (24.8%)	3 (3.2%)	
Candore, G. [21]	94 (76.4%)	24 (19.5%)	5 (4.1%)	122 (80.3%)	26 (17.1%)	4 (2.6%)	
Berlin, D. [12]	67 (67%)	29 (29%)	4 (4%)	66 (66%)	30 (30%)	4 (4%)	
Sampietro, M. [24]	85 (79.4%)	20 (18.7%)	2 (1.9%)	74 (74.7%)	24 (24.3%)	1 (1%)	
Robson, KJ. [25]	138 (72.2%)	50 (26.2%)	3 (1.6%)	194 (72.1%)	67 (24.9%)	8 (3%)	
**Total**	404	129	14	523	170	20	0.96

**Table 6:** Meta-analysis of the C282Y variation in the three published studies regarding Parkinson's disease

**PD**	C282Y						
	**Patients**			**Controls**			

**Author**	wt/wt	wt/mut	mut/mut	wt/wt	wt/mut	mut/mut	*p*

Dekker, M. [10]	125 (91.2%)	10 (7.3%)	2 (1.5%)	2616 (89.7%)	290 (10%)	8 (0.3%)	
Dekker, M. [10]	54 (90%)	6 (10%)	0	2616 (89.7%)	290 (10%)	8 (0.3%)	
Buchanan, D. [11]	391 (89.3%)	46 (10.5%)	1 (0.2%)	405 (83.5%)	76 (15.7%)	4 (0.8%)	
Borie, C. [20]	66 (93%)	5 (7%)	0	53 (91.4%)	5 (8.6%)	0	
**Total**	636	67	3	3074	371	20	0.55

**Table 7:** Meta-analysis of the H63D variation in the two published studies regarding Parkinson's disease

**PD**	H63D						
	**Patients**			**Controls**			

**Author**	wt/wt	wt/mut	mut/mut	wt/wt	wt/mut	mut/mut	*p*

Dekker, M. [10]	104 (76%)	31 (22.6%)	2 (1.4%)	2185 (75%)	661 (22.7%)	68 (2.3%)	
Dekker, M. [10]	44 (73.3%)	16 (26.7%)	0	2185 (75%)	661 (22.7%)	68 (2.3%)	
Borie, C. [20]	42 (63.6%)	23 (34.8%)	1 (1.5%)	39 (66.1%)	20 (33.9%)	0	
**Total**	190	70	3	2224	681	68	0.21

## References

[B1] Bomford A (2002). Genetics of haemochromatosis. The Lancet.

[B2] O'Neil J, Powell L (2005). Clinical Aspects of hemochromatosis. Semin Liver Dis.

[B3] Le Gac G, Ferec C (2005). The molecular genetics of haemochromatosis. Eur J Hum Genet.

[B4] Casetta I, Govoni V, Granieri E (2005). Oxidative Stress, Antioxidants and Neurodegenerative Diseases. Curr Pharm Des.

[B5] Zhu X, Lee HG, Casadesus G, Avila J, Drew K, Perry G, Smith MA (2005). Oxidative imbalance in Alzheimer's disease. Mol Neurobiol.

[B6] Moreira PI, Siedlak SL, Aliev G, Zhu X, Cash AD, Smith MA, Perry G (2005). Oxidative stress mechanisms and potential therapeutics in Alzheimer disease. J Neural Transm.

[B7] Pulliam JF, Jennings CD, Kryscio RJ, Davis DG, Wilson D, Montine TJ, Schmitt FA, Markesbery WR Association of HFE mutations with neurodegeneration and oxidative stress in Alzheimer's disease and correlation with APOE. Am J Med Genet B Neuropsychiatr Genet.

[B8] Kaur D, Andersen J (2004). Does cellular iron dysregulation play a causative role in Parkinson's disease?. Ageing Res Rev.

[B9] Jenner P (2003). Oxidative stress in Parkinson's disease. Ann Neurol.

[B10] Dekker MC, Giesbergen PC, Njajou OT, van Swieten JC, Hofman A, Breteler MM, van Duijn CM (2003). Mutations in the hemochromatosis gene (HFE), Parkinson's disease and parkinsonism. Neurosci Lett.

[B11] Buchanan DD, Silburn PA, Chalk JB, Le Couteur DG, Mellick GD (2002). The Cys282Tyr polymorphism in the HFE gene in Australian Parkinson's disease patients. Neurosci Lett.

[B12] Berlin D, Chong G, Chertkow H, Bergman H, Phillips NA, Schipper HM (2004). Evaluation of HFE (hemochromatosis) mutations as genetic modifiers in sporadic AD and MCI. Neurobiol Aging.

[B13] American Psychiatric Association (1994). Diagnostic and Statistical Manual of Mental Disorders, 4th revised edition.

[B14] McKhann G, Drachman D, Folstein M, Katzman R, Price D, Stadlan EM (1984). Clinical diagnosis of Alzheimer's disease: report of the NINCDS-ADRDA Work Group under the auspices of Department of Health and Human Services Task Force on Alzheimer's Disease. Neurology.

[B15] Folstein MF, Folstein SE, McHugh PR (1975). Mini-Mental State: A practical method for grading the cognitive state of patients for the clinician. J Psychiatr Res.

[B16] Morris JC (1993). The Clinical Dementia Rating (CDR): Current version and scoring rules. Neurology.

[B17] Petersen RC, Smith GE, Waring SC, Ivnik RJ, Tangalos EG, Kokmen E (1999). Mild cognitive impairment: clinical characterisation and outcome. Archives of Neurology.

[B18] Hughes AJ, Daniel SE, Kilford L, Lees AJ (1992). Accuracy of clinical diagnosis of idiopathic Parkinson's disease. A clinico-pathological study of 100 cases. JNNP.

[B19] Saunders AM, Strittmatter WJ, Schmechel D, George-Hyslop PH, Pericak-Vance MA, Joo SH, Rosi BL, Gusella JF, Crapper-MacLachlan DR, Alberts MJ (1993). Association of apolipoprotein E allele epsilon 4 with late-onset familial and sporadic Alzheimer's disease. Neurology.

[B20] Borie C, Gasparini F, Verpillat P, Bonnet AM, Agid Y, Hetet G, Brice A, Durr A, Grandchamp B (2002). Association study between iron related genes polymorphisms and Parkinson's disease. J Neurol.

[B21] Candore G, Licastro F, Chiappelli M, Franceschi C, Lio D, Rita Balistreri C, Piazza G, Colonna-Romano G, Grimaldi LM, Caruso C (2003). Association between the HFE mutations and unsuccessful ageing: a study in Alzheimer's disease patients from Northern Italy. Mech Ageing Dev.

[B22] Moalem S, Percy ME, Andrews DF, Kruck TP, Wong S, Dalton AJ, Mehta P, Fedor B, Warren AC (2000). Are hereditary hemochromatosis mutations involved in Alzheimer disease?. Am J Med Genet.

[B23] Combarros O, Garcia-Roman M, Fontalba A, Fernandez-Luna JL, Llorca J, Infante J, Berciano J (2003). Interaction of the H63D mutation in the hemochromatosis gene with the apolipoprotein E epsilon 4 allele modulates age at onset of Alzheimer's disease. Dement Geriatr Cogn Disord.

[B24] Sampietro M, Caputo L, Casatta A, Meregalli M, Pellagatti A, Tagliabue J, Annoni G, Vergani C (2001). The hemochromatosis gene affects the age of onset of sporadic Alzheimer's disease. Neurobiol Aging.

[B25] Robson KJ, Lehmann DJ, Wimhurst VL, Livesey KJ, Combrinck M, Merryweather-Clarke AT, Warden DR, Smith AD (2004). Synergy between the C2 allele of transferrin and the C282Y allele of the haemochromatosis gene (HFE) as risk factors for developing Alzheimer's disease. J Med Genet.

